# Experimental Evaluation of UWB Indoor Positioning for Indoor Track Cycling

**DOI:** 10.3390/s19092041

**Published:** 2019-05-01

**Authors:** Kevin Minne, Nicola Macoir, Jen Rossey, Quinten Van den Brande, Sam Lemey, Jeroen Hoebeke, Eli De Poorter

**Affiliations:** IMEC, IDLab, Department of Information Technology, Ghent University, 9000 Ghent, Belgium; nicola.macoir@ugent.be (N.M.); jen.rossey@ugent.be (J.R.); quinten.vandenbrande@ugent.be (Q.V.d.B.); sam.lemey@ugent.be (S.L.); jeroen.hoebeke@ugent.be (J.H.)

**Keywords:** ultra-wideband (UWB), indoor localization, tracking, indoor track cycling

## Abstract

Accurate radio frequency (RF)-based indoor localization systems are more and more applied during sports. The most accurate RF-based localization systems use ultra-wideband (UWB) technology; this is why this technology is the most prevalent. UWB positioning systems allow for an in-depth analysis of the performance of athletes during training and competition. There is no research available that investigates the feasibility of UWB technology for indoor track cycling. In this paper, we investigate the optimal position to mount the UWB hardware for that specific use case. Different positions on the bicycle and cyclist were evaluated based on accuracy, received power level, line-of-sight, maximum communication range, and comfort. Next to this, the energy consumption of our UWB system was evaluated. We found that the optimal hardware position was the lower back, with a median ranging error of 22 cm (infrastructure hardware placed at 2.3 m). The energy consumption of our UWB system is also taken into account. Applied to our setup with the hardware mounted at the lower back, the maximum communication range varies between 32.6 m and 43.8 m. This shows that UWB localization systems are suitable for indoor positioning of track cyclists.

**Dataset:** http://dx.doi.org/10.17632/fkhfjfspkr.1

## 1. Introduction

Accurate sports localization has gained much interest during the last couple of years [1]. Using localization, athletes can optimize their performance and coaches can better assist their athletes. Localization systems can be applied during training and competition. Today, there are already many commercially-available radio frequency (RF)-based solutions for sports localization. However, none of them are applied in indoor track cycling.

The subject of the research presented in this section is the positioning of track cyclists and the ideal mounting location for the UWB hardware. ZigBee-based solutions for accurate indoor localization of track cyclists were proposed and evaluated in [2,3]. In [4], a particle filter was designed for tracking a fast-moving bicycle in an indoor velodrome using inertial sensors and infrequent position measurements. This has the downside that it requires timing measurements on the track; a camera-based system was used for this.

In [5,6], many applications of UWB were highlighted. Ultra-wideband has many advantages compared to other localization technologies. UWB can provide good accuracy; it is immune to multipath fading; and it has low power consumption [7]. Because of the many advantages of a UWB system, it looks very promising to use this technology for tracking cyclists in an indoor velodrome.

In this paper, the feasibility of tracking cyclists with UWB technology will be investigated. The main focus of this research is to determine the optimal position of the UWB hardware on the bicycle or cyclist and the position of the infrastructure hardware. The UWB hardware that was mounted on the bike/cyclist was used to position the cyclist, and is called the tag from now on. The infrastructure UWB hardware that needs to be installed in the velodrome allows the tag to calculate its own position. We call this hardware anchors from now on.

A static measurement was performed to evaluate six different tag locations. In indoor track cycling, two different types of bikes were used: a track bike and a pursuit bike. The posture of the cyclist on the track bike was different from his/her posture on the pursuit bike. Therefore, the tag positions were evaluated for both postures. The influence of the anchor height was evaluated by testing the tag position for two different anchor heights. Next to accuracy, the comfort of wearing an UWB tag is also important. The tag should not interfere with the performance and normal movement of the cyclist. In this paper, we rated the comfort of the different tag positions based on the advice of a semi-professional cyclist. Next to finding the optimal tag position, the energy consumption was evaluated, and the maximum communication range was determined.

The main contributions of this paper are as follows:

- The field evaluation of received power (RP) level, accuracy, number of line-of-sight (LOS) anchors, and the open area communication range for different tag positions on bike and cyclist, considering different postures, different anchor heights, and the comfort of the cyclist.
- Evaluation of the energy consumption of the utilized hardware.
- All measured data and results were made publicly available [8].

The remainder of the paper is organized as follows: In Section 2, related work is discussed. In the first part, related research for accurate localization in sports is mentioned, followed by a brief overview of relevant related research in UWB localization. The hardware that was used to evaluate the different tag positions can be found in Section 3. Section 4 outlines the setup of the static measurement. Section 5 is dedicated to the evaluation of the different tag positions, the evaluation of the energy consumption, and the determination of the maximum communication range. Possibilities for future research are mentioned in Section 6. This paper ends with the conclusions in Section 7.

## 2. Related Work

### 2.1. Accurate Localization in Sports

Currently, UWB is barely considered in the specific use case of localization of track cyclists. At the moment, many cycling tracks only record lap times via a system from Mylaps [9]. A transponder is mounted on the fork of the bike, and detection loops are installed under the track. The detection loops are connected to a decoder, which registers the time when a transponder crosses a loop. On an online platform [10], lap times are updated lap by lap, so a coach can follow a session of the cyclist in real time. By visualizing the ridden line of the cyclist(s), it becomes possible to optimize team performance during team pursuit. In team pursuit, cyclists follow their teammates closely in line, drafting to minimize total drag. Periodically, the lead rider peels off the front, swings up the track banking, and rejoins the group at the rear. Riders spend much time trying to optimize this action. The goal is to make sure that the rider who will rejoin the team does not end up to far behind the group. If he/she does this action perfectly, a minimum amount of energy is needed. By using an UWB tracking system, the path of the riders can be visualized, and this can help to optimize the performance of the riders.

An UWB localization system was evaluated on an athlete in [11]. In the first part, static and dynamic measurements were performed to find the optimal tag position. The following positions were investigated: front and side of the right arm and upper legs, chest, neck, belly, head, and hip. They found that the optimal tag position was the head, followed by the neck. In the second part, a particle and Kalman filter were tested for the localization of a cyclist on an indoor track. Anchor nodes were placed on the railing of the track, and the UWB tag was mounted on the helmet of the cyclist. They found that both algorithms showed similar performance; a benefit of the particle filter is that non-Gaussian noise models and a sports-specific path or constraints are easily added. They obtained an accuracy of around 20 cm with the tag placed on the head. In their research, the optimal tag position on the cyclist was not evaluated.

In [12], a quality assessment of a commercial available UWB positioning system was made for athlete tracking during indoor wheelchair court sports. They achieved an average horizontal positioning error of 0.37 m and found that the tag update rate and the number of wheelchairs on the court did not have a significant impact on the positioning quality. In addition, different tag positions were investigated, and the best results were obtained when the tag was mounted in a global positioning system (GPS) vest.

The validation of another commercially-available UWB positioning system was the subject of the study in [13]. They investigated the system during linear and change-of-direction running drills on an indoor court. The researchers evaluated total distance, mean and peak speed, and mean and peak acceleration. They obtained a mean bias in the range of 0.2–12%, with errors between 1.2 and 9.3%.

In [14], the researchers showed that a commercially-available UWB system can efficiently be used to show the increase in muscular capacity. They analyzed the performance of a professional athlete during his recovering time after surgery. The tests were performed on an indoor and outdoor football field with the UWB tag positioned on the shoulder of the athlete.

UWB-based solutions are not the only systems that are currently applied in sports localization. Solutions that use other RF technologies for tracking athletes exist; these use the global navigation satellite system (GNSS), Bluetooth Low Energy (BLE), and Wi-Fi technology. GNSS-based systems use signals from satellites in space to determine the location of the athlete. This solution is not suited for indoor localization of track cyclists. In [15], two GPS systems (10-Hz and 18-Hz GPS) and one UWB solution (20 Hz) were evaluated. They measured the distances covered and the mechanical properties of athletes during sprints on an outdoor sports-specific circuit. The UWB system outperformed both GPS systems: the ultra-wideband solution had better reliability and validity. BLE and Wi-Fi also use fixed position infrastructure hardware like UWB instead of satellites in space. In general, the accuracy of BLE and Wi-Fi solutions is lower than UWB solutions. In [16], the performance of a commercial BLE-based localization system for use in basketball was evaluated. They found an average root mean squared error (RMSE) for all distances and velocities measured of 0.30 cm. Next to RF-based localization systems, there are also vision-based systems. These systems tend to be more accurate (mm accuracy can be provided) [17], but come at a higher cost. Another drawback is due to the inherent nature of camera-based systems: the cyclist needs to be in the line-of-sight of multiple cameras. This can be solved by installing more cameras, which results in even higher costs. Two different types of camera-based motion capture systems exist, optoelectronic measurement systems (OMS) and image processing systems (IPS). The first category of systems determines the 3D location of placed markers on the athletes. Image processing systems do not require markers; the captured images are digitally analyzed to locate the athletes. In [18], an OMS was used to track an athlete during alpine skiing. They obtained sufficient accuracy, but the solution has limited practical usability because of the small capture volume and the obscuration of the markers due to snow spraying.

Much research has already been done in the field of accurate localization in sports. Some researchers already applied localization technology for the use case of indoor track cycling. However, none of them evaluated the optimal position of a UWB tag to track the cyclist in terms of signal quality and comfort.

### 2.2. Ultra-Wideband Localization

The ultra-wideband localization technology is not only used in sports, but in many different sectors. In [19], the accuracy of such a system was evaluated for real-time localization of dynamic resources on construction sites. They showed that the accuracy was inversely proportionate to the velocity of the tag, the number of tags being used, and the complexity of the path on which the tag is moving. In addition, they also investigated the optimal tag location on a worker. The researchers found that a tag mounted on top of the helmet of the worker had the highest accuracy.

The indoor path loss model of ultra-wideband localization has been the topic of many research papers. In [20], a path loss formula based on the narrowband two-path model was proposed and validated with indoor experiments. The path loss for UWB indoor localization was also the subject of the research paper [21]. A measurement campaign was setup, and typical indoor scenarios like line-of-sight (LOS) and non-line-of-sight (NLOS) were investigated. In our research, the maximum communication range of the different tag positions was calculated based on the maximum allowable path loss.

Another important aspect of localization is energy consumption. The influence of different UWB physical settings on the energy efficiency and robustness of communications was investigated in [22]. In this research, the energy consumption of the setup is theoretically calculated.

## 3. Hardware

### 3.1. Wi-Pos System

**Figure 1:**
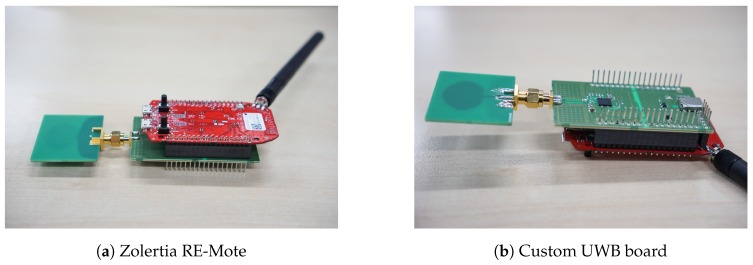
Hardware.

The Wi-Pos system is an in-house-designed UWB research platform. Wi-Pos is used for the tag and anchors, and it consists of the Zolertia RE-Mote Internet of Things (IoT) platform [23], together with a custom UWB shield [24]. The hardware is shown in Figure 1. The Zolertia RE-Mote supports two low power IoT radios (CC1200 and CC2538). In this paper, only the CC1200 sub-GHz radio was used. Both boards were connected trough a serial peripheral interface (SPI) bus. The UWB shield housed the Decawave DW1000 UWB transceiver. The Decawave DW1000 chip was chosen because it is widely available, it has good accuracy, and a comprehensive data sheet is available. An external UWB antenna was connected to the UWB board via a subminiature Version A (SMA) connector.

### 3.2. UWB Antenna

**Figure 2:**
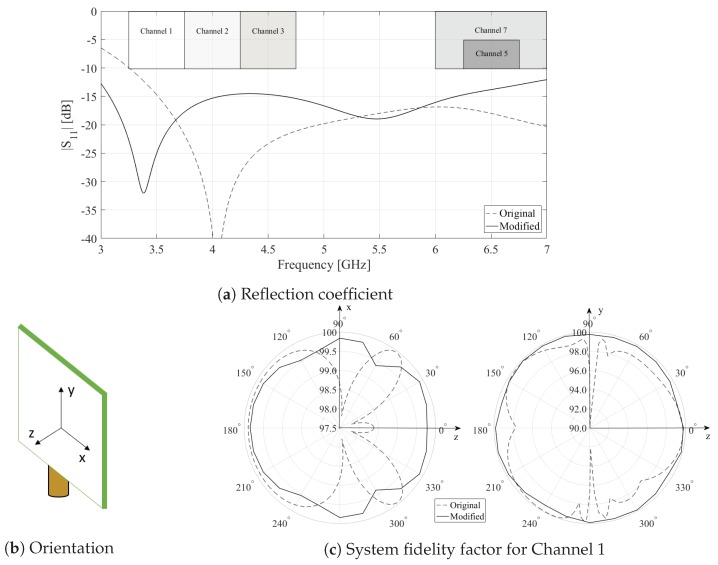
Comparison between the standard monopole antenna proposed by Decawave and the modified version.

In this work, a modified version of the standard monopole antenna proposed by Decawave [25] was used. The modified version was optimized for a four-layer IS400 [26] printed circuit board (PCB) implementation, with the radiating antenna element and ground plane on the two inner copper layers. As a result, the monopole antenna was sandwiched between two IS400 layers, providing a more robust antenna operation in realistic deployment scenarios. During the measurements, Channel 1 of the IEEE 802.15.4a standard [27] was used. As such, the modified antenna was optimized for a system fidelity factor (SFF) higher than 98% for an input pulse of Channel 1, to guarantee minimal antenna-induced ranging errors [28]. As both the anchor and tag nodes used the same antenna, the transmit and receive antenna in the SFF optimization process were chosen to be identical. The simulated reflection coefficient ($S_{11}$), w.r.t. 50Ω, of the standard and modified antenna is shown in Figure 2a as a function of frequency. The simulated SFF of both the standard and modified antenna is shown in Figure 2c. In order to indicate the antenna orientation in the SFF plots, a 3D representation of the modified antenna is provided in Figure 2b with the corresponding Cartesian coordinate system. It is clear that the modified antenna outperformed the standard monopole antenna proposed by Decawave in Channel 1, for both $S_{11}$ and SFF.

## 4. Test Setup

**Figure 3:**
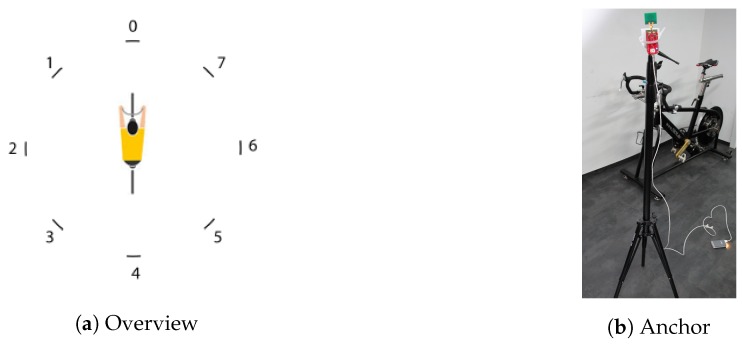
Setup.

To find the optimal tag position, a static measurement was performed. For accurate 3D localization, a tag needs to determine the range with respect to at least four anchors. On an indoor cycling track, there are multiple options to install the anchors. Anchors can be mounted on the ceiling, on the railing that encloses the track, and on tripods that can be placed on the central square or in the tribune behind the railing. In order to cover all possible anchor installation situations, the setup shown in Figure 3a was used to evaluate the different tag positions. Eight anchors were placed on a circle with a radius of two meters (numbered from 0–7). The bike with the test person was placed in the center of this circle. The anchors were mounted on height-adjustable tripods, shown in Figure 3b. In the first set of tests, the height of the anchors was 1.5 m. A second anchor height of 2.3 m was also tested. Two different bikes were used, a track bike and a pursuit bike. Anchors and tags were powered with battery packs.

**Figure 4:**
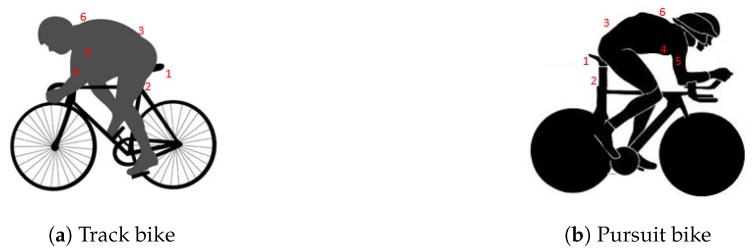
The different tag positions.

In total, six different tag positions were considered; these positions for both bikes are shown in Figure 4 and are listed below:

1. Underneath the saddle
2. Seat post
3. Lower back
4. Chest
5. Upper arm
6. Upper back

**Figure 5:**
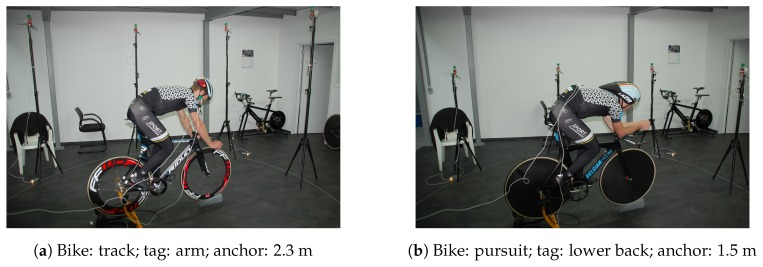
Static measurement.

The pursuit bike had two disc wheels. On the pursuit bike, the cyclist wore a special aerodynamic helmet, which was longer than a standard helmet. A picture of the test with the track bike, the tag secured on the upper arm, and anchor height equal to 2.3 m is shown in Figure 5a. A picture of the test with the pursuit bike, the tag secured on the lower back, and an anchor height of 1.5 m is shown in Figure 5b.

## 5. Evaluation

The performance of the different tag positions was evaluated on different aspects. Accuracy, received power level, number of line-of-sight anchors, comfort, maximum communication range, and energy consumption were investigated. The used UWB radio settings are shown in Table 1. The low data rate and high preamble length resulted in a long range. The highest possible pulse repetition frequency was chosen to obtain higher accuracy on the first path time stamp. The downside of this choice was that this required greater power consumption. All raw data of the tests, as well as all constructed graphs to visualize the measured signals and the calculated values are available in the related dataset [8].

### 5.1. Accuracy

**Figure 6:**
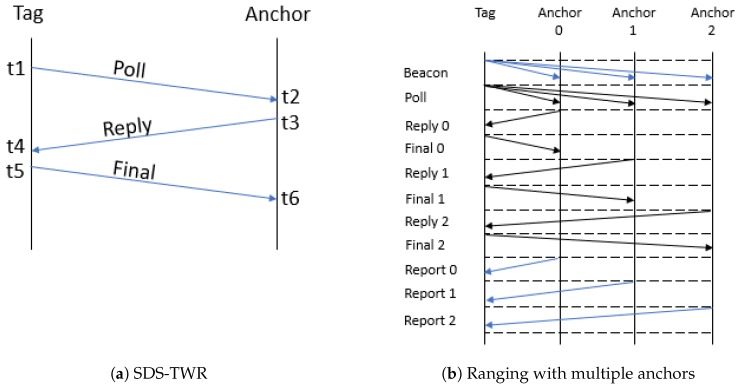
Ranging. SDS-TWR, symmetric double-sided-two way ranging.

The range between the tag and an anchor was calculated based on symmetric double-sided-two way ranging (SDS-TWR). Three UWB messages were exchanged to calculate a range between the tag and anchor: poll, reply, final. The message exchange is shown in Figure 6a. This way of ranging has the advantage of eliminating clock drift. The range can be calculated using: range=ToF.c, with *c* equal to the speed of light. The time of flight (ToF) was calculated with Formula (1), where the variables are related to the timestamps used in Figure 6a.

(1) $$ToF=\frac{\left(t4-t1)(t6-t3)-(t3-t2)(t5-t4\right)}{\left(t4-t1)+(t6-t3)+(t3-t2)+(t5-t4\right)}$$

The ranges between the tag and the eight anchors were read from the serial output of the tag. Ranging with multiple anchors was done sequentially, starting with Anchor 0 till Anchor 7. In total, 100 samples per anchor per test were collected. The communication procedure for the ranging with three anchors is shown in Figure 6b. It started with the tag broadcasting a beacon using sub-GHz (blue) containing the set of anchors with which to determine the range, followed by the tag broadcasting a poll to the set of anchors. Each anchor replied sequentially and received the final message from the tag. In the end, the ranges were broadcast from the anchors using sub-GHz. The tag received and printed this range.

#### 5.1.1. Absolute Ranging Error

**Figure 7:**
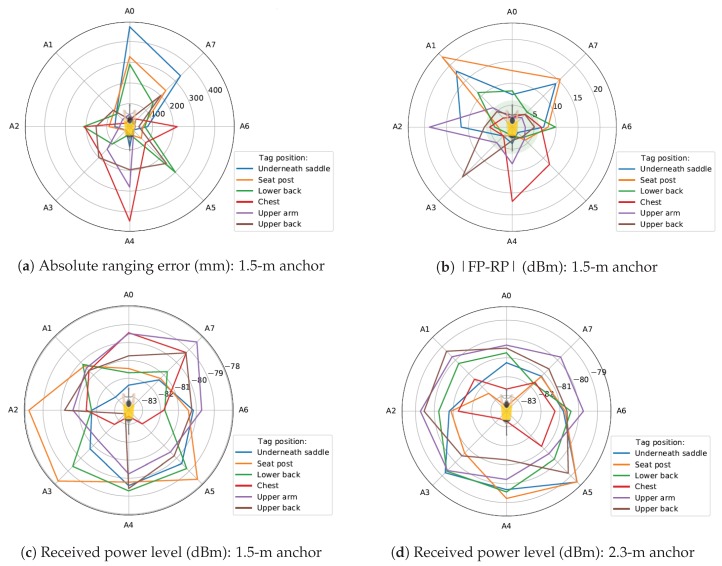
Results tests: track bike.

To be able to calculate the absolute ranging error, the ground-truth is required. The ground-truth was determined with a mm-accuracy laser meter. The absolute error to each anchor for six different tag positions is plotted in Figure 7a. The cyclist was placed on the track bike, and the anchors were mounted at a height of 1.5 m. From this plot, it was clear that the ranging for the tag positions underneath the saddle and seat post to the anchors in front (Anchors A1, A0, and A7) were the least accurate. When the tag was placed on the chest of the cyclist, the ranging to the anchor behind the cyclist (anchor four) was less accurate. For the upper arm tag position, Anchors 3 and 4 had the greatest absolute ranging error. Except for Anchor 5 and 7, the error was smaller than 200 mm when the tag was placed at the upper back. The greatest error for a lower back-mounted tag was for Anchor 0 and Anchor 5. The median values of the absolute ranging error to each anchor for all tests can be found in the related dataset [8].

#### 5.1.2. Cumulative Distribution Function of the Ranging Error

**Figure 8:**
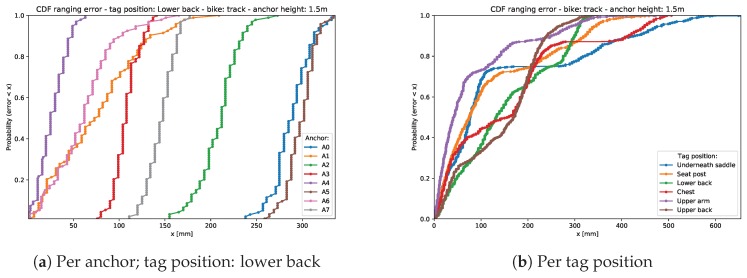
Example CDF ranging error; bike: track; anchor height: 1.5 m.

The cumulative distribution of the ranging error per anchor was plotted on the same figure, and an example is shown in Figure 8a. In this example, the tag was placed on the lower back. Anchor 4 had the smallest error, and this was the closest anchor.

To be able to compare different tag positions, the absolute errors to each anchor were aggregated per test. Figure 8b gives the cumulative distribution of the ranging error for six different tag positions. This plot was made for both bikes and both anchor heights and can be found in the related dataset [8]. For the setup used in Figure 8b, the upper arm tag position had the highest probability to have a small ranging error.

### 5.2. Received Power Level

Figure 7c shows the measured received power (RP) level of each anchor for six different tag positions, again for the track bike and an anchor height of 1.5 m. We remark that the center of this plot had the smallest received power level, so the ideal tag position had a received power level plot that was a wide circle.

**Figure 9:**
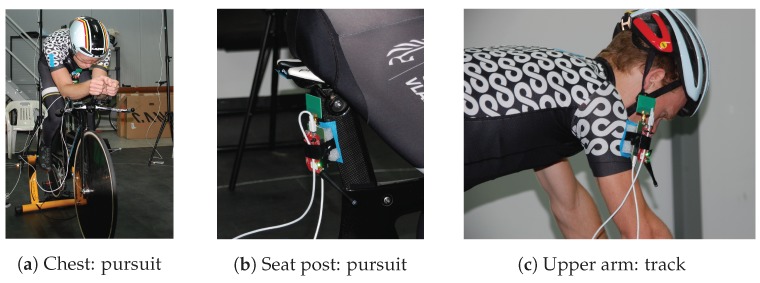
Different tag positions on different bikes.

The worst tag position was the chest, as the signal was blocked by the cyclist and the RP level for Anchors A3, A4, and A5 was very low. The human body influences propagation behavior by reflecting and absorbing (for the most part) the radiated waves [29]. The same blocking behavior was noticeable when the tag was mounted on the bike, tag positions being underneath the saddle and seat post. A seat post-mounted tag on the pursuit bike is shown in Figure 9b. Now, the RP level of the anchors in front of the cyclist was low. Figure 7d shows the RP level for an anchor height of 2.3 m. For the chest tag position, the RP level of anchor 0 decreased if the anchor height increased, and this was due to bad antenna orientation. The RP level of Anchor 0 was even lower if the cyclist was on the pursuit bike at this anchor height. This setup is shown in Figure 9a. It can be seen that the antenna orientation was bad, and the antenna radiated to the body and to the ground. The chest tag was also enclosed by the body of the cyclist.

Increasing the anchor height did not always have a bad influence on the received power level. When the tag was mounted on the lower back of the cyclist, the performance actually improved and when anchors were mounted higher. The received power level of Anchors 2 and 6 increased. The RP level of this tag position for the pursuit bike was similar to the track bike, which was good. This means that this tag position was suitable for races with the track and the pursuit bike.

We now evaluate the upper arm tag position; the tag was mounted on the right upper arm of the cyclist, shown in Figure 9c. Anchors A0, A7, and A6 have the highest RP level when anchors are mounted at 1.5 m, which is the logical result of the chosen setup. For an anchor height of 2.3 m, the behavior changed: the RP level of the anchors on the left of the test person increased. This position also had a similar performance for the pursuit bike.

The last tag position that is discussed is the upper back. For the track bike and an anchor height of 1.5 m, the RP level of Anchor 3 was the smallest power level of all considered tag positions. This is not to be expected, and probably, something went wrong with this measurement. In the next section, the criteria for an anchor to be in the line-of-sight is discussed. It will become clear that Anchor 3 is non-line-of-sight. This behavior was not observed for the pursuit bike and at a higher anchor height. Because of above reasons, it was assumed that this RP level was the consequence of a software problem. Neglecting Anchor 3, the behavior of this tag position with anchors mounted at 2.3 m was similar to the lower mounted anchors.

### 5.3. Line-of-Sight Detection

We want to determine the number of anchor nodes that are in the line-of-sight and the number of anchors that are not in the line-of-sight during a test. This gives an indication of the level of blocking by the cyclist or by the bike. The following rule of thumb was used [30]:

If the absolute value of the difference between the first path power (FP) level and the received power (RP) level was smaller than or equal to 6 dBm, the anchor was likely to be in the line-of-sight (LOS). If this value was greater or equal to 10 dBm, the anchor node was likely to be in the non-line-of-sight (NLOS). If the difference lied between 6 and 10 dBm, we did not know if the anchor was LOS or NLOS.

The RP and FP values were captured during the static measurement described in Section 4, therefore, this difference can easily be calculated. An overview of the number of LOS, NLOS, and undetermined anchors of all measurements can be found in the related dataset [8]. The value of the absolute difference between the first path and received power level for the setup with the track bike and anchors mounted at 1.5 m is depicted in Figure 7b. The green zone on this plot indicates a value smaller than or equal to 6 dBm.

A table that gives an overview of this absolute difference for all measurements can be found in the related dataset [8]. From this table, it can be deduced that only three configurations had all eight anchors’ LOS, and these are shown in Table 2. When the tag was mounted on the lower back of the cyclist and anchors were mounted at a height of 2.3 m, all anchors were in the line-of-sight, independent of the type of bike that was used.

Figure 7b shows the result when anchors were mounted at a height of 1.5 m and the track bike was considered. The tag position that had the most LOS anchors was the upper back; seven anchors were line-of-sight, and one anchor was non-line-of-sight. This was already mentioned in the section about the received power level.

The blocking behavior of the bike and cyclist for tag positions underneath the saddle and seat post was confirmed by this absolute value. For all considered anchor heights and bikes, Anchors A1, A0, and A7 were never LOS. These anchors were even always NLOS for the seat post tag position. The blocking of the signal by the body of the cyclist for the chest tag position was also confirmed: Anchor 4 was always NLOS for all measurements. The worst situation was a chest-mounted tag, track bike, and anchors at 2.3 m; in this case, there were no LOS anchors.

### 5.4. Comfort

The comfort of the cyclist wearing the UWB hardware was taken into account. It is possible that a tag position has excellent performance on received power level, the number of LOS anchors, and accuracy, but that this position is not suitable to use in practice because the tag hinders the athlete. The tracking system is to be used during training and competition. If the tag has a negative impact on the performance of the cyclist, it is very likely that the cyclist will refuse to use the system. The measurements happened with a semi-professional cyclist, who is currently the performance analyst of the Belgian national indoor track cycling team. With his advice, the different tag positions were given a comfort rating, shown in Table 3. A score of five equals very comfortable, and zero is very uncomfortable.

From the ratings above, it is clear that the upper back position was the most uncomfortable. This is because of the setup with the pursuit bike. In this case, the cyclist wore a special aerodynamic helmet, shown in Figure 5b. This helmet had a long tail, and this tail hindered the tag. Because this aerodynamic helmet was always used when the cyclist rode the pursuit bike, this tag position was not suitable.

The most comfortable tag positions were the ones where the tag was mounted on the bike. The second most comfortable position was the lower back. The tag can easily be mounted here because there is a small pocket in the skin suit that riders wear during training and competition.

### 5.5. Maximum Communication Range

The possible communication range of a system is an important factor when analyzing the feasibility. Based on this, the number of necessary anchors for the enrollment of the tracking system can be determined. We remark that an indoor cycling track is an open area with no obstacles between tags and anchors. In this section, the maximum communication range, assuming an open area between tags and anchors, is discussed. The sensitivity power level determines the power level after which the packet cannot be detected or decoded anymore. By adapting the modulation setting, the sensitivity level can be changed [31]. The maximum communication range can be increased by increasing the transmission power.

**Figure 10:**
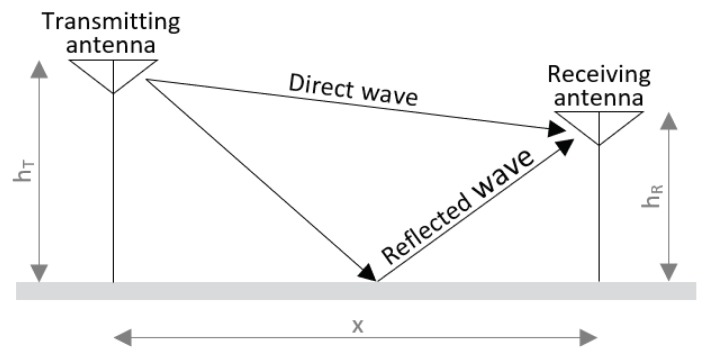
Two-path model.

We now consider the two-path propagation model shown in Figure 10 [20]. This model assumes a direct wave between the tag and anchor and a reflected wave from the planar ground. It takes interference between these two waves into account, and the total received energy is the vector sum of the direct and the reflected wave. The difference in path length will result in distances where both signals will interfere destructively and distances with constructive interference.

The difference between transmitted and received power is called path loss. The path loss fluctuates significantly when the distance between tag and anchor is short, and the envelope of the path loss is then proportional to the square of the distance (n=2). When the distance between tag and anchor is much greater than the height of the tag and anchor, the path loss increases in proportion to the fourth power of the distance (n=4). The boundary between these two regions is called the breakpoint $D_{b}$ and can be calculated with Formula (2).

(2) $$D_{b}=\frac{2\pi h_{T}h_{R}}{\lambda }$$

where λ is the wavelength, $\lambda =\frac{c}{f}$. The center frequency *f* for Channel 1 was equal to 3494.4 MHz and *c* equal to the speed of light. If we calculate the breakpoint distance for anchor and tag height $h_{T}=h_{R}=1.5$ m, we get a breakpoint distance $D_{b}=164.78$ m.

The path loss (dB) at distance *d* can be calculated with Equation (3). It consists of the path loss measured at a reference distance $d_{0}=2$ m and a logarithmic term on the relative increase in distance between transmitter and receiver. Further, it also takes shadowing into account by the term $X_{\sigma }$.

(3) $$PL=TP-RP=PL_{0}+10n\log_{10}\left(\frac{d}{d_{0}}\right)+X_{\sigma }$$

where:

TP= transmitted power in dBm
RP= received power in dBm
$PL_{0}=$ path loss at reference distance in dB
n= path loss exponent
d= distance in m, $d>d_{0}$
$d_{0}=$ reference distance in m
$X_{\sigma }=$ shadowing term ∼N(0,σ)

The transmitted power by the Decawave DW1000 chip Channel 1 was −14.32 dBm [32]. The path loss exponent *n* was equal to two because the considered distances were smaller than the calculated breakpoint distance $D_{b}=164.78$ m.

**Figure 11:**
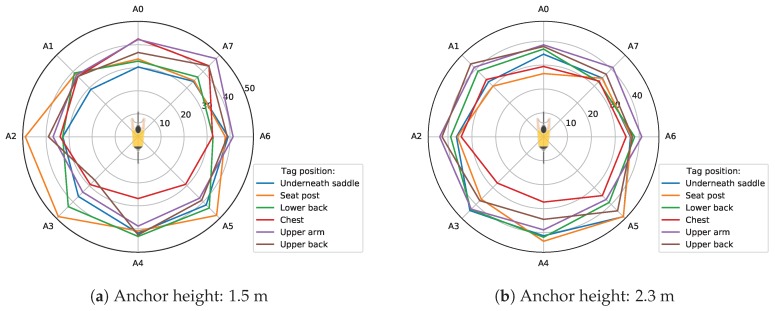
Maximum communication range (m); bike: track.

The maximum possible communication range from the tag to each anchor can be calculated using the measured RP levels. The minimum received power that was required for reliable ranging was −106 dBm, and the transmitted power was equal to −14.32 dBm, which results in a maximum allowable path loss of 91.68 dB. The path loss at the reference distance was equal to the difference between transmitted power and the measured received power. The maximum communication range can now be found using Formula (3) (remember, n=2 and $d_{0}=2$ m). The results of these calculations for each anchor and for each possible configuration of tag, bike, and anchor heights were appended to the related dataset [8]. These results were plotted on polar plots and can also be found in the related dataset [8]. Figure 11a,b show the maximum communication range to each anchor for the track bike and both anchor heights.

When the tag was mounted on the bike, the maximum communication range to the anchors in front of the bike was the lowest for both anchor heights. A chest-mounted tag achieved the greatest communication range to the anchors in front. The maximum range of tag positions lower back, upper back, and upper arm approximated a circular pattern for an anchor height of 2.3 m. Considering these three tag positions, the upper arm tag had the greatest range to the anchor in front, and the lower back tag had the greatest range to the anchor behind the cyclist.

For each combination of tag position, bike, and anchor height, the minimum and maximum value of the calculated maximum communication ranges to the eight anchors were determined. These results can be found in the table: Overview of the maximum ranges, which is available in the related dataset [8]. These values were aggregated per tag position, and the result is shown in Table 4. A similar conclusion as the one that followed from the received power level can be drawn. For tag positions underneath the saddle and seat post, the maximum communication range achieved a minimal value for an anchor in front of the rider. For the chest position, this value was the smallest for the anchor behind the rider. This is also noticeable in Figure 11a,b. A second conclusion is that the difference between the smallest and largest value of the maximum communication range was the smallest for the lower back tag position.

### 5.6. Energy Consumption

The energy consumption of the solution is another aspect that needs to be taken into account. Because the tag is mounted on the cyclist or on the bike, it needs to be battery powered. For indoor track cycling, a tag that can work for four consecutive hours is sufficient. This is because training and competition almost never exceed four hours. An anchor has a fixed location, for example mounted on a tripod, and can be connected to the electrical grid. Battery-powered anchors have the advantage of an easy and quick setup because no cables are required. In general, the power consumption of an anchor can be much lower than the power consumption of a tag, and this will become clear in the following paragraphs.

In [33], the energy consumption of an anchor of our hardware and software solution was already discussed. The hardware consisted of the Decawave DW1000 UWB transceiver [31] and a CC1200 sub-GHz radio [34]. The difference between our solution and a traditional UWB system is that a traditional approach always listens to the UWB radio. In our solution, the anchors can set their UWB radio in sleep-mode while they are not selected by the tag. They only listen to the beginning of the superframe for the synchronization message using the sub-GHz radio. The current consumption of a stand-by anchor was 3.4 mA. When an anchor is selected to determine the range, it will only power on the UWB radio on the slots on which they are supposed to range. The current consumption of an active anchor was 26.6 mA. In comparison, a traditional approach consumes on average of 130 mA. This means that our solution had a significant current reduction on the anchor nodes. The current consumption of the tag will be much higher than the current consumption of an anchor as the tag is constantly ranging with different anchors.

In [24], the current consumption of the tag was measured. Furthermore, the average current consumption of a tag and anchor node was calculated. When the tag is powered by a 6000-mAh battery pack, the tag can be powered for 74 h. A powered anchor with this battery pack can last for 99 h, which again shows that a tag consumes more energy than an anchor.

### 5.7. Summary of the Main Results

Six different tag positions were evaluated on two different bikes. Different anchor heights were considered, namely 1.5 and 2.3 m. Table 5 gives the results for an anchor height of 1.5 m. The tag positions are ordered on the number of LOS anchors and received power level. The column “Comfort” tries to rate how comfortable the tag position was. A score of five means very comfortable, zero very uncomfortable. For each measured value, the 50th and 90th percentiles are given. The column “Max.” range gives an overview of the lowest and highest value for the calculated maximum communication range to the respectively worst and best anchor per tag position. Table 6 gives the same results, but for an anchor height of 2.3 m.

From these two tables, the following can be concluded. The tag position upper back had the most LOS anchors for both anchor heights. The chest position performed well for an anchor height of 1.5 m, but had a dramatic performance for an anchor height of 2.3 m with zero LOS anchors. The value of the maximum communication range to the best possible anchor decreased from 43.599 m to only 34.766 m. For an anchor height of 1.5 m, three tag positions had five LOS anchors, and based on the RP level and ranging error, we concluded that the tag positions chest and upper arm had a similar performance. The lower back performances increased if the anchor height increased. The ranging error decreased, and the number of LOS anchors and RP level increased. However, the maximum communication range to the best and worst possible anchor stayed approximately the same. Tag positions on the bike, seat post, and underneath the saddle performed poorly for both anchor heights.

Three tag positions were uncomfortable for the cyclist; therefore, the tag cannot be mounted on the upper back, the upper arm, and chest. The signal from the tag positions on the bike, underneath the saddle, and seat post was disturbed by the bike and cyclist. The signal from the tag mounted on the chest was also disturbed by the cyclist. Therefore, these three tag positions are not suitable. The tag position that had an acceptable performance level (especially at higher anchor heights) and was comfortable for the cyclist was the lower back. For our use case, this tag position outperformed the other positions.

From the above results, it is clear that a larger anchor height improves the performance of the lower back-mounted tag. Ceiling-mounted anchors are preferred. An important remark is that the vertical and horizontal dilution of precision (VDOP and HDOP) values of the setup should be taken into account. A low value for the VDOP and HDOP is of great importance for the performance of the localization system. If all anchors are mounted at the same height above the velodrome, the VDOP value will be very large. Therefore, a combination of ceiling-mounted anchors and anchors on the central square mounted on tripods should be installed. This will prevent all anchors being in the same plane and will improve the localization accuracy.

## 6. Future Work

Investigating the signal quality of a setup in a real-life scenario where anchors are mounted in an indoor velodrome, in combination with a tag mounted on the lower back can be the subject of future research for indoor tracking of cyclist based on UWB signals. A dynamic measurement can be performed to find the optimal radio settings of the UWB chip. The optimal localization algorithm should also be determined, and the performance of different antennas can be investigated.

## 7. Conclusions

In this research, the different UWB tag positions on a cyclist were evaluated in order to obtain accurate localization of the athlete during training and competition on an indoor cycling track. A static measurement was performed, and six different tag positions were evaluated in four different setups. Due to a different posture of the cyclist on different bikes, a track and a pursuit bike were used. The influence of the anchor height was investigated by considering two different anchor heights. The tag positions were evaluated based on accuracy, received power level, number of line-of-sight anchors, and comfort. The upper back tag position had the most LOS anchors for both considered anchor heights. However, this tag position is very uncomfortable for the cyclist, as this position hinders the cyclist on the pursuit bike. When anchors are placed at 2.3 m, the lower back tag position has the second highest number of LOS anchors. This position has an acceptable performance level (especially at higher anchor heights) and is comfortable for the cyclist. Next to the optimal tag position, also energy consumption and maximum communication range were investigated. It was shown that the energy consumption of our hardware is limited. For the lower back tag position, the maximum communication range varied between 32.6 m and 43.8 m in the considered setup. This shows that UWB localization systems are suitable for indoor positioning of track cyclists.

References

## Figures and Tables

**Table 1:** UWB radio settings.

Parameter	Value
Channel	1
Data rate	110 kbps
Pulse repetition frequency (PRF)	64 MHz
Preamble length	1024 symbols

**Table 2:** Overview configurations with all anchors’ LOS.

Measurement	Bike	Anchor Height (m)	Tag Position
9	Track	2.3	Lower back
12	Track	2.3	Upper back
21	Pursuit	2.3	Lower back

**Table 3:** Comfort ratings of different tag positions.

	Underneath Saddle	Seat Post	Lower Back	Chest	Upper Arm	Upper Back
**Comfort Rating**	5	5	4	2	2	0

**Table 4:** Range (m) for all tag positions.

Tag Position	Min. Range		Max. Range	
	Range (m)	Anchor	Range (m)	Anchor
Underneath saddle	29.106	A1	48.549	A5
Seat post	26.430	A0	49.038	A2
Lower back	32.563	A7	43.758	A5
Chest	23.987	A4	43.599	A7
Upper arm	33.992	A4	48.437	A6
Upper back	26.624	A3	43.773	A5

**Table 5:** Overview of the results; anchor height: 1.5 m.

Order	Tag Position	Comfort	Ranging Error (mm)		RP Level (dBm)		LOS		Max. Range (m)	
			50%	90%	50%	90%	50%	90%	Min.	Max.
1	Upper back	0	23	37	−80.74	−80.38	6	6	26.624	43.479
2	Chest	2	26	40	−79.45	−79.27	5	5	23.987	43.599
3	Upper arm	2	49	68	−79.50	−79.28	5	5	33.992	48.437
4	Lower back	4	290	313	−81.69	−81.02	5	5	32.586	43.758
5	Seat post	5	327	360	−81.45	−80.48	4	4	29.557	49.038
6	Underneath saddle	5	471	554	−82.38	−81.78	3	3	29.106	43.599

**Table 6:** Overview results: anchor height: 2.3 m.

Order	Tag Position	Comfort	Ranging Error (mm)		RP Level (dBm)		LOS		Max. Range (m)	
			50%	90%	50%	90%	50%	90%	Min.	Max.
1	Upper back	0	43	62	−80.49	−80.15	8	8	32.533	43.773
2	Lower back	4	220	269.5	−80.73	−80.52	6	6	32.563	43.755
3	Upper arm	2	37	61	−80.33	−80.02	5	5	36.718	43.899
4	Underneath saddle	5	151	199.5	−81.26	−81.03	4	4	30.425	48.549
5	Seat post	5	805	838	−83.58	−82.21	3	3	26.430	48.409
6	Chest	2	431	487	−82.66	−82.10	0	0	26.539	34.766
